# Efficacy of sellar opening in the pituitary adenoma resection of transsphenoidal surgery influences the degree of tumor resection

**DOI:** 10.1186/s12880-017-0217-5

**Published:** 2017-07-24

**Authors:** Shousen Wang, Yong Qin, Deyong Xiao, Liangfeng Wei

**Affiliations:** Department of Neurosurgery, Fuzhou General Hospital, Fujian Medical University, No. 156 Xi’erhuanbei Road, Fuzhou, 350025 People’s Republic of China

**Keywords:** Sellar opening, Hypopituitarism, Pituitary adenoma, Transsphenoidal surgery, Degree of tumor excision, Complication

## Abstract

**Background:**

Endonasal transsphenoidal microsurgery is often adopted in the resection of pituitary adenoma, and has showed satisfactory treatment and minor injuries. It is important to accurately localize sellar floor and properly incise the bone and dura matter.

**Methods:**

Fifty-one patients with pituitary adenoma undergoing endonasal transsphenoidal microsurgery were included in the present study. To identify the scope of sellar floor opening, CT scan of the paranasal sinus and MRI scan of the pituitary gland were performed for each subject. Intraoperatively, internal carotid artery injury, leakage of cerebrospinal fluid, and tumor texture were recorded, and postoperative complications and residual tumors were identified.

**Result:**

The relative size of sellar floor opening significantly differed among the pituitary micro-, macro- and giant adenoma groups, and between the total and partial tumor resection groups. The ratio of sellar floor opening area to maximal tumor area was significantly different between the total and partial resection groups. Logistic regression analysis revealed that the ratio of sellar floor opening area to the largest tumor area, tumor texture, tumor invasion and age were independent prognostic factors. The vertical distance between the top point of sellar floor opening and planum sphenoidale significantly differed between the patients with and without leakage of cerebrospinal fluid.

**Conclusion:**

These results together indicated that relatively insufficient sellar floor opening is a cause of leading to residual tumor, and the higher position of the opening and closer to the planum sphenoidale are likely to induce the occurrence of leakage of cerebrospinal fluid.

## Background

Pituitary adenoma [1], tumors at in the pituitary gland, represent from 10% to 25% of all intracranial neoplasms, and the estimated prevalence rate in the general population is approximately 17% [2, 3]. In the clinical practice, neurosurgeons commonly adopted endonasal transsphenoidal microsurgery for the resection of pituitary adenoma, which showed satisfactory treatment effects with minor injuries. However, accurately identifying the intraoperative localization of sellar floor is always challenging in the clinical practice, due to the limited space in the surgical operation, anatomical variations of sphenoid sinus, as well as its adjacent structures, including conchal sphenoidal sinus, sphenoidal sinus separation (e.g., multiple, horizontal or slanting), flat sellar floor, and Onodi cell. Inaccurate identifications of sellar floor would induce the unsuccess of the operation [4]: (1) if the localization of sellar floor deviated frontally, it would occur error entries into the posterior ethmoidal sinus and tuberculum sellae; (2) if the localization of sellar floor deviated bilaterally, it would probably lead to the injuries of the cavernous segment of the internal carotid artery (CSICA), as well as the injuries of cranial nerves in cavernous sinus, thus resulting in intraoperative massive hemorrhage, and several post-operation clinical symptoms, e.g., ambiopa; (3) if the localization of sellar floor deviated posteriorly, it would cause injuries to the brain stem. Thus, it has been proposed to develop individualized approach to identify the location of sellar floor based on the three-dimensional reconstruction (3-DR) of the relevant anatomical landmarks in the operation, with advantages of allowing for simplifying the process, improving the direction sense, increasing the positioning accuracy, and shortening the operation time.

With the precise localization of sellar floor, it is of vital importance to properly incise the bone and dura matter, to guarantee the satisfactory treatment of the endonasal transsphenoidal microsurgery for the resection of pituitary adenoma. Conventionally, the scope of the sellar floor opening in the endonasal transsphenoidal microsurgery would not exceed the tuberculum sellae and the sellar- clivus point frontally and posteriorly respectively, and close to the medial wall of the cavernous sinus. Abe et al. [5] applied the bone window CT scanning parallel to the transsphenoidal approach to help determine the lateral boundaries of sellar opening, which showed good operative performance. However, these are generalized boundaries and scopes across patients, it still remains unclear about individualized sellar floor opening procedure, as well as the potential factors affecting the sellar floor opening.

The majority of studies discussing the influencing factors of pituitary adenoma excision focused on the clinical characterizations of the tumor [6–8], e.g., the size, texture, invasiveness and orientation of the tumor, with the factors regarding the scope and positioning of sellar floor opening remaining unclearly. Indeed, Mattozo et al. [9] and Alahmadi et al. [10] conducted transsphenoidal excision of the residual pituitary adenoma under microscope and endoscope, and concluded that insufficiency of sphenoidal sinus anterior wall and sellar floor opening of the previous surgery are the major causes of residual tumor. However, several drawbacks existed in these studies leading to the inaccurate conclusions, including (1) they were descriptive studies based on the subjective experience of the researchers, without the solid demonstrations; (2) with the long intervals between the two consective surgeries, there may be the enlargement and migration of the residual tumors, as well as bone ossifications of the anterior wall of the sphenoid sinus and sellar defects. In addition, Wei et al. [11] reported that excessive sellar floor opening probably causes injury of pituitary gland and leakage of cerebrospinal fluid. Wang et al. [12] hypothesized that larger sellar floor opening enlarged the surgical field, aggravated mechanical irritation to adjacent structures, and led to the incidence of posterior pituitary gland injury and diabetes insipidus.

Based on these understanding, the present study aims to comprehensively and quantitatively assess the relationship between sellar floor opening (scope and position) and intraoperative/postoperative complications (e.g., leakage of cerebrospinal fluid, diabetes insipidus and hypopituitarism).

## Methods

### Patients

Fifty-one patients ((22 males and 29 females, aging from 19 to 75 years with average of 46.7 ± 12.6 years) with pituitary adenoma, undergoing endonasal transsphenoidal surgeries in the Department of Neurosurgery of Fuzhou Genera Hospital from March 2014 to March 2015 were included in the present study. Inclusion criteria were (1) complete medical data including preoperative and postoperative CT scan of paranasal sinus, and MRI of pituitary gland, hormone levels at preoperative 3 days and postoperative 1 week; (2) all surgeries completed by one single physician; (3) all clinical data provided by one single medical center (i.e., Fuzhou Genera Hospital); (4) the diagnosis of pituitary adenoma validated by immunohistochemical staining. Exclusion criteria were (1) a history of pituitary adenoma surgery or radiotherapy; (2) a history of sphenoidal sinus trauma, surgical trauma or malignant tumor.

According to the clinical data of patients, 44 patients suffered from headache, 21 patients with visual field defects, 2 patients with acromegaly, and 10 patients with amenorrhea-galactorrhea syndrome. Physical examination revealed that 1 patient presented declining sexual function, 1 patient with stuffy nose, 1 patient with palpitation, and 1 patient with chest distress. According to the immunohistochemical staining analysis [13], 13 patients with null-cell adenoma, 15 patients with prolactin (PRL) adenoma, 1 patient with growth hormone (GH) adenoma, 11 patients with plurihormonal adenoma, 7 patients with gonadotropic tumor, 1 patient with thyroid stimulating hormone (TSH) adenoma, and 3 patients with adrenocorticotropic hormone (ACTH) adenoma. According to the tumor diameter, the patients were divided into the micro-, macro-, and giant-adenoma groups [14], which were respectively defined as follow: (1) micro-adenoma with tumor diameter less than 10 mm; (2) macro-adenoma with tumor diameter between 10 and 30 mm; (3) giant-adenoma with tumor diameter larger than 30 mm. As a result, 2 patients had microadenoma, 35 patients had macroadenoma, and 14 patients had giant adenoma.

### CT and MRI scan

At 1 week before and after surgeries, CT plain scan of paranasal sinus was performed separately using 256-slice spiral CT scanner (Discovery Ultra, GE Corporation). Scan parameters were consecutive scan along with the axis position, layer thickness of 0.625 mm with an interval of 0.625 mm, scan time of 1.2 s, FOV of 250 mm × 250 mm, and matrix of 512 × 512. All patients received CT scan in a supine position. Infraorbital line was regarded as the base line. Scan area ranged from the mandible to the upper frontal sinus. With multiple planar reconstruction (MPR) reconstructed using CT reconstruction software, the three-dimensional image models of the sella turcica were calculated in the environment of Mimics 15.0 software (Materialise Inc., Belgium).

**Fig. 1:**
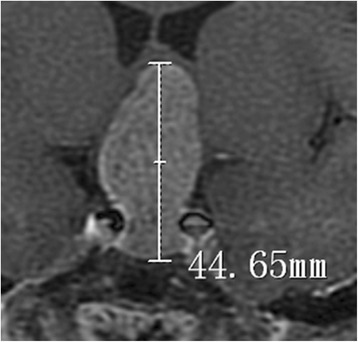
Schematic diagram of the maximal tumor diameter evaluated by enhanced coronal MRI (44.65 mm represents the maximum diameter of this pituitary adenoma)

At 1 week before and after surgeries, T1WI, T2WI, and T1WI contrast-enhanced MRI was performed separately using 3.0 T MRI system (Trio Tim, SIE corporation). MRI parameters were: layer thickness of 1.0 mm, FOV 250 mm × 250 mm, matrix 256× 256 and scan time of 6 min. Contrast-enhanced MRI was performed via intravenous injection of Gd-DTPA at a dosage of 0.2 mmol/kg body weight at a flow speed of 3.6 ml/s. Using INFINITT software (Seoul, South Korea), the localization of tumor were identified from the adjacent tissues. The maximal tumor diameter and the tumor area (S) of each layer were calculated automatically in coronal position. All tumors were divided into micro-, macro- and giant –adenoma (as illustrated in Fig. 1), and the tumor volume was calculated [15].

Based on the method proposed by Knosp et al. [16] the degree of tumor invasiveness was classified into grades from 0 to 4: (1) grade 0, the tumor margin did not pass the tangent of the medial aspects of the supra- and intracavernous ICA; (2) grade 1, the tumor margin did not pass a line between the cross-sectional centers of the carotid arteries, the so-called “intercarotid line”; (3) grade 2, the tumor margin extended beyond the intercarotid line, but does not extend beyond or tangent to the lateral aspects of the intra- and supracavernous ICA; (4) grade 3, the tumor margin extended lateral to the lateral tangent of the intra- and supracavernous ICA; (5) grade 4, the CSICA was completely encased by tumor tissues. If the Knosp classification grade higher than 3, the tumors were defined as the invasive pituitary adenoma.

### Evaluation of pituitary gland function by measuring blood levels of hormones

Hormone levels were measured at 8 a.m. of 2 days after admission and postoperative 7 days. The venous blood samples were collected for chemiluminescence analysis (Siemens ADVIA Centaur XP automatic electrochemical luminescence analyzer, Germany). The hormones of GH, ACTH, free triiodothyronine (FT3), free thyroxine (FT4), estradiol (E2), testosterone (T), TSH, PRL, follicle stimulating hormone (FSH), luteotropic hormone (LH), cortisol and insulin-like growth factor 1 (IGF-1), were measured and summarized in Table 1.

**Table 1:** Normal reference values of hormone levels

Hormones	Reference value
GH	<10 ng/mL for females, <1 ng/mL for males
ACTH	4.7-48.8 pg/mL
Cortisol	Blood sampling in the morning: 4.3-22.4 g/dL
	Blood sampling in the afternoon: 3.09-16.66 g/dL
FT3	3.5-6.5 pmol/L
FT4	11.5-22.7 pmol/L
TSH	0.35-5.5 μ IU/mL
FSH	2.5-10.2 mIU/mL at follicular stage; 3.4-33.4 mIU/mL at ovulatory stage
	1.5-9.1 mIU/mL at luteal stage; 23-116 mIU/mL at menopause stage
LH	1.9-12.5 mIU/mL at follicular stage; 8.7-76.3 mIU/mL at ovulatory stage
	0.5-16.9 mIU/mL at luteal stage; 15.9-116 mIU/mL at menopause stage
E2	15.9-144.2 pg/mL at follicular stage; 63.9-356.7 pg/mL at ovulatory stage
	55.8-214.2 pg/mL at luteal stage; 0-32.2 pg/mL at menopause stage
T	14-76 ng/dL
IGF-1	127-584 ng/mL for 18-20 years old; 116-358 ng/mL for 21-30 years old
	109-307 ng/mL for 31-40 years old; 94-267 ng/mL for 41-50 years old
	81-238 ng/mL for 51-60 years old; 69-212 ng/mL for 61-70 years old
PRL	2.5-10.2 mIU/mL at follicular stage; 1.8-20.3 ng/mL at ovulatory stage
	1.5-9.1 mIU/mL at luteal stage; 1.8-20.3 ng/mL at menopause stage
	2.8-29.2 ng/mL for non-pregnant women; 9.7-208 mIU/mL for pregnant women

Patients were diagnosed as hypopituitarism for hypofunction of anyone axis, such as pituitary gland-thyroid axis, growth hormone axis, adrenal cortex axis or gonad axis [17]: (1) hypofunction of adenohypophysis-gonad axis were identified as testosterone level lower than normal range for male patients; serum FSH/LH lower than normal range for female patients at menopause; decreased level of estradiol accompanied by normal or decreased levels of FSH and LH for the female at non-menopause, amenorrhoea or infrequent menstruation [5]; (2) hypofunction of adenohypophysis-thyroid axis were identified as serum level of FT4 lower than normal range accompanied by low or normal TSH level; (3) hypofunction of adenohypophysis-adrenal cortex axis were identified as cortisol level lower than normal reference value and normal ACTH or lower than normal reference value [18].

### Surgical procedures

The surgical procedures via right single nostril-sphenoidal sinus approach, performed and recorded under camera-equipped surgical microscope, were completed by one single physician as follows. After disinfection, the cotton strips, soaked with 0.0067% adrenaline saline were, inserted into bilateral nasal cavities for 3 min, for nasal vasoconstriction and nasal cavity dilatation. An incision was made in the nasal septum (approximately 3 cm posterior to anterior naris). The surgical approach extended to the anterior wall of sphenoidal sinus between the right periosteum and perpendicular plate of ethmoid bone, with bilateral ostia of the sphenoid sinus exposed and the anterior wall of sphenoidal sinus removed. According to CT and MRI data of preoperative paranasal sinus, the position of sella turcica was comprehensively determined based upon the vomer, Onodi cell, sphenoidal sinus separation, and sellar protuberance. In addition, the regime of sellar floor opening was individualized designed according to tumor size, sella turcica, sphenoidal sinus, minimal distance between bilateral CSICA, etc. Following sellar floor opening, the opening scope of dura mater was determined to match with the bone opening as possible. With the resected tumor specimen prepared for pathological examination, the tumorous cavity was packed with gelatin sponge, fixed and sealed by medical glue. Then, one drainage tube of sphenoidal sinus was retained, and bilateral nasal cavities were filled with vaseline gauze. At postoperative 1 day, the drainage tube was removed and the gauze was abandoned at 3 days after surgery. Intraoperatively, the incidence of ICA injury, leakage of cerebrospinal fluid and tumor texture were recorded for each patient. All tumor tissues were divided into the hard and soft tumor groups according to the criteria proposed by Yamamoto et al. [19].

### Postoperative evaluations

The average amount of urine per hour and 24 h were recorded after surgery. Diagnostic criteria of diabetes insipidus: specific gravity of urine lower than 1.005; consecutive 2-h amount of urine more than 600 ml or amount of urine more than 2500 ml/d; requiring antidiuresis medication. All these criteria should be properly met when identified as diabetes insipidus. The endocrine secretion of pituitary gland was re-examined at the 7th postoperative day. The plasma levels of electrolytes were measured on a regular basis. CT scan of the paranasal sinus was conducted to identify the incidence of intracranial hemorrhage.

**Fig. 2:**
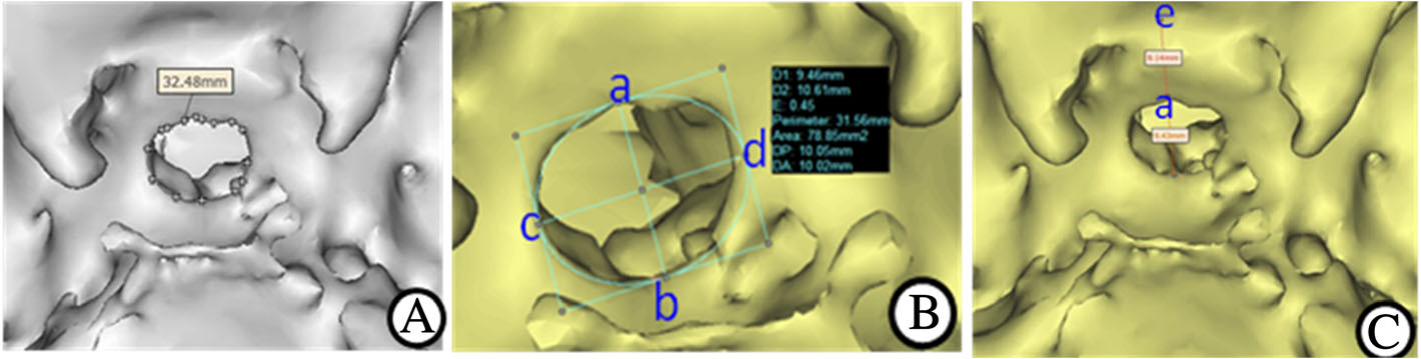
CT 3-DR images revealing postoperative morphology of sellar floor and relevant parameters. **a**. postoperative morphology of sellar floor; **b**. ab = 9.45 mm as the maximal vertical diameter of sellar floor opening, cd = 10.62 mm as the maximal transverse diameter of sellar floor opening, Area: 78.85 mm^2^ as the approximate opening area; **c**. ae = 9.14 mm as the vertical distance between the top point of sellar opening and planum sphenoidale

Multiple planar reconstruction was conducted after CT scan by using the Mimics 15.0 software (Materialise corporation, Belgium). Three-dimensional images of the sella turcica were calculated and constructed and then subject to dynamic partitioning. The bone opening area was calculated according to the equation as below. Area = π × (maximal transverse diameter/2) × (maximal vertical diameter/2) = π/4 × maximal transverse diameter × maximal vertical diameter. The plane with maximal vertical diameter was selected to quantitatively measure the vertical distance between the top point of sellar floor opening and planum sphenoidale, as illustrated in Fig. 2.

According to the MRI scanning, the incidence, size, and position of residual tumor were identified. Specifically, the degree of tumor excision was evaluated by calculating the ratio of preoperative to postoperative tumor size. For total tumor resection, no residual tumor could be noted by enhanced-MRI examination. For sub-total resection, the percentage of residual tumor size should be less than 10%, and 10%-40% for partial excision of the tumors [20].

To evaluate the effects of sellar floor opening, appropriate sellar floor opening was defined as no tumor residues were detected by postoperative MRI for patients undergoing total resection surgery. For the patients with residual tumors, appropriate sellar floor opening was defined if residual tumors were located in anterior cranial fossa, posterior cranial fossa or in the cavernous sinus, whereas insufficient opening was identified for those with residual tumors inside the sella.

If postoperative imaging reveals the residual suprasellar tumor, CT image of the paranasal sinus should be fused with MRI image of the pituitary gland, for the reconstruction of the sellar floor opening and residual tumor. In combination with tumor texture and morphology, comprehensive evaluations were made. If tumor texture was hard and/or in dumb-bell shape, sellar floor opening was defined as appropriate. If tumor texture was soft and wasn’t in dumb-bell shape, sellar floor opening was defined as insufficient, with the tumor resected by enlarging sellar floor opening. If the degree of tumor excision cannot be enhanced by widening sellar floor opening, sellar floor opening was defined as appropriate.

All the surgical specimens were sent to the Department of Pathology of the medical center for pathological examination. Tumor tissues were fixed into 10% neutral formalin solution, paraffin embedding and sections for H.E staining, as well as staining of Syn, PRL, GH, ACTH, TSH, FSH, LH, etc. Positive outcome was obtained if above 10% of the cells were stained according to the diagnostic criteria of pituitary adenoma proposed by the WHO in 2004 [20].

### Statistical analysis

The measured data, mentioned above, were analyzed in the SPSS 17.0 statistical software (SPSS Inc., Chicago, IL). Particularly, normally-distributed measurement data were expressed as mean ± standard deviation, whereas non-normally-distributed data were expressed as median + range interquartile (M + QR). To identify the differences between groups, qualitative data were statistically analyzed by chi-square test or Fisher exact test, whereas quantitative data were analyzed by independent sample t-test or Mann-Whitney U test. Linear relationship between two variables was analyzed by Pearson correlation analysis. Moreover, multivariate analysis was performed by logistic regression model. In these statistical analysis, *P* < 0.05 was considered as statistical significance.

## Results

### Tumor characteristics

Across the 51 patients, the course of diseases ranged from 3 days to 20 years (2.5 ± 4.0 years in average), the tumor diameter ranged from 0.8 to 4.3 cm, with an average of 2.4 ± 0.9 cm. The maximal tumor area in coronal position was measured in the range from 0.2 to 10.7 cm^2^, with an average of 4.2 ± 2.6 cm^2^. The tumor volume was in the range from 0.2 to 30.2 cm^3^, with an average of 7.4 ± 6.8 cm^3^. The morphology of tumor was summarized as follow: 12 patients with lobulated tumor, 2 patients with dumbbell-shaped tumor, and 37 patients with oval-shaped tumor. The invasiveness of the tumor was summarized as follow: 17 patients with Knosp classification grade 0, 7 patients with grade 1, 11 patients with grade 2, 9 patients with grade 3, and 7 patients with grade 4. The deviations of tumor were summarized as follow: 24 patients with midline deviations, 11 patients with left deviations, 16 patients with right deviations. In addition, there were 9 patients with MRI T2WI hypointense signal, 42 patients with isointense signal, and 15 patients complicated with cystic lesions in the tumor.

### Intraoperative evaluations

For all patients, preoperative 3D CT reconstruction images were consistent with intraoperative observations of sphenoidal sinus separation. The sellar floor was rapidly localized under the guidance of sphenoidal sinus separation. No obvious complications induced by localization deviation were observed. Intraoperatively, 8 patients showed hard tumor texture, and 44 patients showed soft tumor texture. In addition, 11 patients presented leakage of cerebrospinal fluid, and no ICA injury was noted.

### Postoperative evaluations

Across the 51 patients, 35 patients received total tumor resection, 9 patients received sub-total resection, and the other 7 patients received partial resection. In terms of residual tumor location, 5 patients had suprasellar tumor, 7 patients with cavernous sinus, 2 patients with suprasellar plus anterior cranial fossa, 2 patients with cavernous sinus and posterior fossa. The postoperative complications of patients were summarized as follow: 2 patients with intracranial hemorrhage, 3 patients with electrolyte disturbance, 17 patients with temporary diabetes insipidus, and 1 patient with cerebrospinal rhinorrhea.

Across the 51 patients, 15 patients with preoperative hypopituitarism were excluded for the following postoperative evaluation of pituitary function. The results of the remaining 36 patients showed that 13 patients developed hypopituitarism, i.e., 9 patients with gonad axis hypofunction alone, 2 patients with thyroid axis hypofunction, and 2 patients with thyroid axis complicated with gonad axis hypofunction, as illustrated in Table 2.

**Table 2:** Postoperative complication of 51 patients undergoing tumor resection

	N (percentages out of 51)
*Degree of tumor resection*	
Total resection	35 (68.6%)
Subtotal resection	9 (17.7%)
Partial resection	7 (13.7%)
*Position of residual tumor*	
Suprasellar region	5 (9.8%)
Cavernous sinus	7 (13.7%)
Suprasellar + anterior cranial fossa	2 (3.9%)
Posterior cranial fossa and cavernous sinus	2 (3.9%)
*Postoperative complication*	
Intracranial hemorrhage	2 (3.9%)
Electrolyte disturbance	3 (5.9%)
Temporary diabetes insipidus	17 (33.3%)
Cerebrospinal rhinorrhea	1 (2.0%)
Hypopituitarism	13 (36.1%)

The area of sellar floor opening was (94.71+ 95.55) mm^2^ vs. (151.00 + 118.38) mm^2^ for the toral tumor resection (35 patients) and residual tumor group (16 patients) respectively, and the area of sellar floor opening was significantly smaller in the total tumor resection group than that in the residual tumor group (*Z* = −2.274, *P* < 0.05). In addition, the ratio of sellar floor opening area to maximal tumor area was 0.44 + 0.35 in the total resection group, significantly larger (*Z* = −2.153, *P* < 0.05) than those in the residual tumor group with ratio of 0.27+ 0.12.

Across the 51 patients, with the extent of tumor resection as dependent variable, and factors including patient’s age, sex, tumor invasion, texture and the ratio of sellar floor opening area to maximal tumor area as independent variables, multi-factor analysis revealed that tumor invasion, tumor texture, age and the ratio of sellar floor opening area to maximal tumor area served can significantly predict the extent tumor resection (*p* < 0.05 for all variables), as illustrated in Table 3.

**Table 3:** Logistic regression analysis of the influential factors of the degree of tumor resection

Factors	B	Standard error	Wald	df	*P*	Exp(B)
Ratio (sellar floor opening area to maximal tumor area)	6.269	2.711	5.348	1	0.021	528.15
Tumor invasion	−2.817	0.969	8.446	1	0.004	0.060
Gender	0.139	0.892	0.024	1	0.876	1.149
Age	−0.093	0.042	5.013	1	0.025	0.911
Tumor texture	−3.823	1.915	3.983	1	0.046	0.022

Across the 51 patients, there were 11 patients presenting cerebrospinal fluid leakage. The vertical distance between the top point of sellar floor opening and planum sphenoidal was summarized as 3.2 ± 2.1 mm vs. 7.9 ± 2.5 mm for the patients with and without cerebrospinal fluid leakage respectively. In addition, there was significant differences between these two groups (*t* = 5.704, *P* < 0.05).

Across the 51 patients, 17 patients suffered from postoperative temporary diabetes insipidus. The sellar floor opening area in the diabetes insipidus group was 157.97 + 166.77 mm^2^, which was not significantly different (*Z* = −1.319, *P* > 0.05) from those patients without diabetes insipidus (112.92 + 78.68 mm^2^).

Across the 51 patients, 36 patients showed normal function of pituitary gland, and 13 patients showed hypopituitarism after surgery. In the hypopituitarism group, the sellar floor opening area was summarized 133.64 + 134.38 mm^2^, which did not significantly differ from (73.79 + 86.85) mm^2^ in their counterparts without hypopituitarism (*Z* = −1.531, *P* > 0.05).

## Discussion

The present study adopted preoperative CT and MRI scan to identify the anatomical variations of sphenoid sinus and adjacent structures, and developed individualized opening strategies combined with the characteristics of tumor and sella turcica, minimal distance of bilateral CSICA and alternative parameters.

The effect of sellar floor opening was explicitly evaluated after the surgery. The majority of the patients displayed appropriate sellar floor opening, and only 2 patients displayed had insufficient sellar floor opening, which negatively influenced the extent of tumor resection. In addition, as revealed by the assessed relationship among multiple variables, i.e., sellar floor opening area, position, degree of tumor resection, intraoperative and postoperative complications, relative insufficiency of sellar floor opening is one of the factors influencing the residual pituitary adenoma. The higher sellar floor opening is more likely to induce the incidence of leakage of cerebrospinal fluid. In contrast, postoperative hypopituitarism is not significantly relevant with sellar floor opening area.

Mattozo et al. [9] and Alahmadi et al. [10] ever reported that sellar floor opening insufficiency is the major cause of residual tumor after the previous surgery. Nevertheless, the possibility of enlargement and migration of the residual tumors also may significantly increase due to relatively long interval between the consective two surgeries. Thus, the conclusion that sellar floor opening is associated with residual tumor, still remain untenable due to lack of solid evidence. Even early signs of errhysis, cataclysm and artificial materials for sellar reconstruction after surgery tend to affect the evaluation accuracy of residual tumors, several research groups [21, 22] insisted no statistical significance noted in terms of the detection rate of residual tumors for patients undergoing MRI between early and late stages. Consequently, postoperative early CT and MRI images were properly fused by two independent experienced physicians, which significantly improved the evaluation accuracy. For tumors extending into the cavernous sinus and anterior cranial fossa, the tumors cannot be fully exposed and resected by conventional microscopic transsphenoidal surgery [23]. For suprasellar residual tumors, although the bone opening has been enlarged, it fails to fully resect the hard and dumbbell-shaped tumors. For such case, sellar floor opening is considered as an appropriate approach.

The sellar floor opening area of the micro- and macro-adenoma groups was significantly smaller compared with that of the giant adenoma group. The sellar floor opening was positively correlated with tumor size, i.e., the larger pituitary adenoma was, the larger the sellar floor opening area was. Moreover, to enhance the exposure and resection of tumors, the sellar floor opening area should be enlarged accordingly. Compared with those in the total resection group, the sellar floor opening area in the sub-total resection group was significantly larger whereas the ratio of sellar floor opening area to maximal tumor area was considerably smaller. Importantly, multi-factor analysis revealed that the ratio of sellar floor opening area to maximal tumor area, tumor texture, invasion and patients’ age act as independent prognostic factors of degree of tumor resection. These results together provide direct evidence that relative insufficiency of sellar floor opening is one of the factors leading to residual tumors.

Since the ratio of sellar floor opening area to maximal tumor area was calculated as (0.44 + 0.35) in the total resection group, it is likely that the total tumor resection can be completed when the ratio of sellar floor opening area to maximal tumor area up to 50%. With the sellar floor opening area excessively small, the degree of tumor resection would be reduced. With the sellar floor opening area excessively large, it is easy to induce ineffective sellar floor opening, resulting in unnecessary injuries. Considering that the limited sample size in the present study, further studies with more samples should be conducted to verify this hypothesis.

The large tumors likely to extending into anterior or posterior cranial fossa and cavernous sinus, cannot be fully exposed and removed by conventional opening via microscopic transsphenoidal approach, while hard tumors located at suprasellar region cannot be completely resected. The results of the present study also indicated that the age of the patients could significantly influence the degree of tumor resection, probably because the tumors in elderly patients are large and have high invasiveness. In consistent with the obtained result in the present study that tumor texture and invasion can significant influence the tumor resection, Losa et al. [24] also proposed that that the cystic lesions of tumors are beneficial for total tumor resection, and several researchers suggest that the orientation of tumor extension and tumor morphology are both important factors of tumor resection.

Across the 51 patients, 2 patients presented small degree of tumor resection, probably resulting from insufficient tumor exposure and mechanical operation. Restricted visual field influences the evaluation on the sinking of diaphragma sella. The suprasellar tumor is mistakenly considered as diaphragma sella or partial sinking of diaphragma sella as complete sinking, leading to residual suprasellar tumors. In addition, compared with that in their counterparts without cerebrospinal fluid leakage, the vertical distance between the top point of sellar floor opening and planum sphenoidale was significantly shorter in patients with leakage of cerebrospinal fluid. It indicates that excessively high sellar floor opening probably is more likely to induce the leakage of cerebrospinal fluid during surgery.

Following the surgeries, approximately 33.3% of enrolled patients developed temporary diabetes insipidus, which is consistent with 15%-70% as reported by previous investigations [25]. As proposed by Wang et al. [26] during transsphenoidal surgery, enlargement of surgical scope is likely to induce injury to the neurohypophysis and its blood supply, eventually leading to the incidence of diabetes insipidus.

Transsphenoidal operation can stimulate or injure adenohypophysis tissues, resulting in the postoperative occurrence of hypopituitarism [15]. Zhou et al. [27] ever reported that 48% of patients presented hypopituitarism induced by surgical procedures. In this study, the incidence rate of hypopituitarism after surgery is 36.1%. Zada et al. [20] showed that male patients with relatively large tumors are more likely to show hypopituitarism after surgery, compared with alternative counterparts. Wei et al. [11] proposed that excessive resection of sellar floor bone probably causes injuries to adenohypophysis, thereby leading to the occurrence of hypopituitarism. Nevertheless, in this investigation, the risk of hypopituitarism is not significantly associated with the size of sellar floor opening.

With preoperative MRI conducted to accurately pinpoint the position of pituitary, intraoperative surgical procedures completed by experienced surgeons experienced in avoiding the risk of excessive traction and averted frequent use of electrocoagulation, the present study provided direct evidence that the enlargement of sellar floor opening effectively increasing the surgical field, allowed for properly distinguishing the tumors from the surrounding normal tissues, but not induce risks of injuries to the pituitary gland, hypothalamus and blood vessels.

There were two weaknesses in this preliminary study. First, in some patients, the residual tumor was located in the cavernous sinus. In clinical practice, some surgeons preferred not to resect tumor in the cavernous sinus. Thus, the residual tumor would be a certain factor if a tumor extends into the cavernous sinuses rather than a result affected by the size of the sella opening. Second, the correlation between the bony opening near the tuberculum sella and CSF leakage was not considered separately for micro and macroadenomas. The sella is likely to have arachnoid in its most rostral part in patients with small tumors, while macroadenomas can completely fill the sella and extend suprasellar; in this circumstance, a more rostral opening of the sellar floor would be much less likely to result in a breach of the arachnoid, and may help with tumor resection. In the future, we will conduct studies to further consolidate our findings by conducting more clinically useful correlation between the extent of bony opening and resection of all “surgically accessible” tumor (i.e., not in the cavernous sinus) and considering separately for micro and macroadenomas.

## Conclusion

This paper indicated that residual tumor could be caused by relatively insufficient sellar floor opening and the occurrence of leakage of cerebrospinal fluid could be induced by choosing the opening position higher and closer to the planum sphenoidale.

## Abbreviations

3-DR: Three-dimensional reconstruction
ACTH: Adrenocorticotropic hormone
CSICA: Cavernous segment of the internal carotid artery
E2: Estradiol
FSH: Follicle stimulating hormone
FT3: Free triiodothyronine
FT4: Free thyroxine
GH: Growth hormone
IGF-1: Insulin-like growth factor 1
LH: Luteotropic hormone
M + QR: Median + range interquartile
MPR: Multiple planar reconstruction
PRL: Prolactin
T: Testosterone
TSH: Thyroid stimulating hormone
